# Comparative genomic analysis of clinical and environmental strains provides insight into the pathogenicity and evolution of *Vibrio parahaemolyticus*

**DOI:** 10.1186/1471-2164-15-1135

**Published:** 2014-12-18

**Authors:** Lei Li, Hin-chung Wong, Wenyan Nong, Man Kit Cheung, Patrick Tik Wan Law, Kai Man Kam, Hoi Shan Kwan

**Affiliations:** School of Life Sciences, The Chinese University of Hong Kong, Shatin, New Territories, Hong Kong SAR, People’s Republic of China; Department of Microbiology, Soochow University, Taipei, 111 Taiwan; Stanley Ho Centre for Emerging Infectious Diseases, JC School of Public Health, Faculty of Medicine, The Chinese University of Hong Kong, Hong Kong SAR, People’s Republic of China; Institute for Molecular Infection Biology, University of Würzburg, Würzburg, Germany

**Keywords:** *Vibrio parahaemolyticus*, Comparative genomics, Clinical, Environment

## Abstract

**Background:**

*Vibrio parahaemolyticus* is a Gram-negative halophilic bacterium. Infections with the bacterium could become systemic and can be life-threatening to immunocompromised individuals. Genome sequences of a few clinical isolates of *V. parahaemolyticus* are currently available, but the genome dynamics across the species and virulence potential of environmental strains on a genome-scale have not been described before.

**Results:**

Here we present genome sequences of four *V. parahaemolyticus* clinical strains from stool samples of patients and five environmental strains in Hong Kong. Phylogenomics analysis based on single nucleotide polymorphisms revealed a clear distinction between the clinical and environmental isolates. A new gene cluster belonging to the biofilm associated proteins of *V. parahaemolyticus* was found in clincial strains. In addition, a novel small genomic island frequently found among clinical isolates was reported. A few environmental strains were found harboring virulence genes and prophage elements, indicating their virulence potential. A unique biphenyl degradation pathway was also reported. A database for *V. parahaemolyticus* (http://kwanlab.bio.cuhk.edu.hk/vp) was constructed here as a platform to access and analyze genome sequences and annotations of the bacterium.

**Conclusions:**

We have performed a comparative genomics analysis of clinical and environmental strains of *V. parahaemolyticus*. Our analyses could facilitate understanding of the phylogenetic diversity and niche adaptation of this bacterium.

**Electronic supplementary material:**

The online version of this article (doi:10.1186/1471-2164-15-1135) contains supplementary material, which is available to authorized users.

## Background

Foodborne diseases remain a serious public health problem worldwide. In Hong Kong, infections due to foodborne pathogens are also a common and important public health issue. Among all causative agents resulting in foodborne outbreaks, bacteria have caused more than 80% of the cases. In 2006, the Centre for Health Protection of Hong Kong revealed more than 800 local foodborne outbreaks due to bacterial causative agents, inflicting more than 3000 people [1]. *Vibrio parahaemolyticus*, a Gram-negative pathogenic halophilic bacterium, is the most prevalent in certain Asian areas and causes food poisoning, occasionally outbreaks, especially in the hot season [2].

*V. parahaemolyticus* is well known as the causative agent of the most prevalent food poisoning in Asia since the mackerel-borne outbreak in 1959 [3]. *V. parahaemolyticus* infections arise from the consumption of raw or undercooked seafood, typically causing gastroenteritis [4]. Infections can become systemic and can be life-threatening to immunocompromised individuals [5].

*V. parahaemolyticus* strains isolated from diarrheal patients produce thermostable direct hemolysin (TDH), TDH-related hemolysin (TRH), or both, while isolates from the environment rarely contain genes encoding these proteins. The TDH is the major virulence gene of *V. parahaemolyticus* and present in most of the clinical Kanagawa phenonemon (KP)-positive strains. In addition to its hemolytic activity, enterotoxicity, cytotoxicity as well as cardiotoxicity have been confirmed in TDH [6]. TDH-related hemolysin (TRH) is produced by KP-negative strains, encoded by *trh* gene, with about 67% amino acid sequence homology and immunologically related with TDH [7]. TRH is hemolytic, heat labile and is suspected to play an unclarified role in the diarrhea caused by these KP-negative strains. After the bacteria produce TDH and lipopolysaccharides released from a dead cell, human host would show strong systemic responses and produce mucosal B-cells against these antigens. However, recent study has also revealed that *V. parahaemolyticus* could profoundly disturb epithelial barrier function in Caco-2 cells with other virulence factors, in the absence of TDH [8]. *V. parahaemolyticus* also encodes two type III secretion systems (T3SS), which are located in different chromosomes. The contribution of these two T3SS has previously been characterized in animal models [9, 10]. These two T3SSs are involved in distinct pathogenic mechanisms during infection. The type III secretion system 1 (T3SS1) is required for cytotoxicity, whereas the type III secretion system 2 (T3SS2) is often responsible for enterotoxicity and intestinal fluid accumulation. The overall mechanism of pathogenesis in *V. parahaemolyticus* remains unclear.

*V. parahaemolyticus* infections are associated with multiple serotypes. Analysis of an outbreak in Calcutta in 1996 has identified a unique serotype clone, O3:K6, which is not previously isolated in that area [11]. After this report, O3:K6 isolates and its serovariants, defined to have identical genotype and molecular characteristics to those isolated in Calcutta, have been reported from foodborne outbreaks across the world. These serotypes were thought to be clonal derivatives of the O3:K6 serotype [12]. Recent multilocus sequence typing (MLST) data have further confirmed that multiple serotypes occur in a single genetic lineage [13].

Comparative genomics analysis of merely clinical strains of *V. parahaemolyticus* was described before [14]. However, the phylogenetic diversity and niche adaptation of *V. parahaemolyticus* at the species level is currently unknown. Strains isolated from environmental reservoirs could be as virulent as clinical strains in animal models [15], and the existing regulatory mechanisms of environmental *V. parahaemolyticus* could favor regulations of foreign virulence genes [16]. Nonetheless, the virulence potential of *V. parahaemolyticus* at the genome-wide level has not been described before. We sequenced the genomes of four O3:K6 and its serovariant clinical strains and five environmental strains of *V. parahaemolyticus* in Hong Kong. Genomes of a few environmental *V. parahaemolyticus* isolates have been announced recently [17–20], however, comparative genomics study has not been reported until now. Phylogenomic analysis of our local strains and other available genomes revealed a clear distinction between clinical and environmental strains.

## Results and discussion

### Summary of genome sequencing data

As summarized in Table 1, we have completed 454 genome sequencing of nine *V. parahaemolyticus* isolates obtained in Hong Kong, including four clinical and five environmental strains. The average sequencing coverage was between 44× to 66×. Genome sequences of these local *V. parahaemolyticus* strains were *de novo* assembled using Newbler 2.7 (Roche Diagnostics). Genome assembly of *V. parahaemolyticus* yielded 130 to 1205 contigs of N50 contig length ranging from 7 kb to 305 kb. The clinical strains we sequenced were new O3:K6 and its serovariant (O3:K59), and they were positive for the *tdh* gene but negative for the *trh* gene. Our environmental strains were all *tdh* and *trh* negative. We have also included in our analyses six publicly available *V. parahaemolyticus* genomes, which are all clinical strains (Table 1) and two of them (AQ3810, AQ4037) pre-pandemic strains [14].

**Table 1:** **List of** ***V. parahaemolyticus*** **clinical and environmental isolates analyzed in this study**

Strain	Specimen collection date	Source	tdh	trh	Serotype	CDS	Reference
VIP4-0395	20/11/07	Local-Stool	+	-	O3:K6	4711	This study
VIP4-0439	10/09/08	Local-Stool	+	-	O3:K6	4733	This study
VIP4-0445	26/09/08	Local-Stool	+	-	O3:K6	6226	This study
VIP4-0407	18/01/08	Local-Stool	+	-	O3:K59	4692	This study
AQ3810	1983	Singapore	+	-	O3:K6	5458	[11]
AQ4037	1985	Maldives	-	+	O3:K6	4447	[9]
RIMD2210633	1996	Thailand	+	-	O3:K6	4831	[12]
Peru-466	1996	Peru	+	-	O3:K6	4603	[9]
AN-5034	1998	Banglandesh	+	-	O4:K68	4770	[9]
K5030	2005	India	+	-	O3:K6	4606	[9]
VIP4-0430	03/07/08	Local-Oyster	-	-	O4:K34	5698	This study
VIP4-0443	23/09/08	Local-Big eye fish	-	-	O2:UT	5994	This study
VIP4-0219	13/04/06	Local-Salmon Sashimi	-	-	O1:UT	4761	This study
VIP4-0444	23/09/08	Local-Big eye fish	-	-	O11:UT	4976	This study
VIP4-0447	22/10/08	Local-Oyster	-	-	O6:UT	5091	This study

UT: Untypeable.

### Phylogenetic relationships

**Figure 1:**
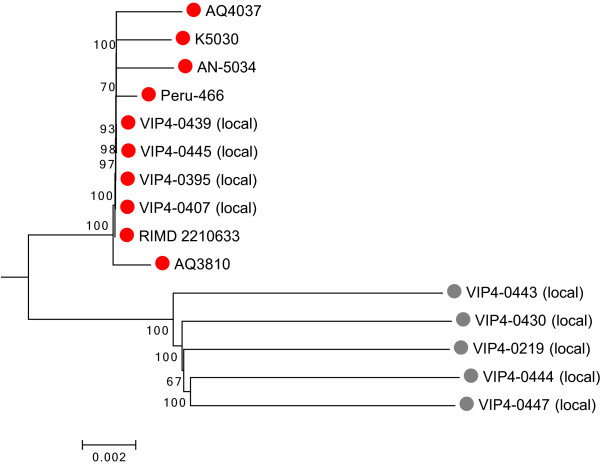
**Neighbor-joining phylogenetic tree showing relationships among** ***V. parahaemolyticus*** **isolates inferred using SNP sites.** Nodal supports were calculated from 500 bootstrap pseudoreplicates. The tree was rooted using *Vibrio harveyi* ATCC BAA-1116. The scale bar represents 0.002 substitutions per nucleotide position. Red color indicates clinical strains, and grey color indicates environmental strains.

Phylogenetic relationships among our *V. parahaemolyticus* isolates and the reference strains (Table 1) were examined using a genome-wide approach based on 169,998 single nucleotide polymorphisms (SNPs). Eighty nine percent of these SNPs were in coding regions, of which 25.8% were missense, 0.6% was nonsense, and 73.6% were synonymous. Genome-wide comparison readily resolved the relationship among the clinical and environmental isolates (Figure 1). Clinical strains were found to be more related compared to the environmental strains. Moreover, our local clinical isolates were highly similar to RIMD 2210633 isolated from Thailand in 1996, consistent with previous results using various molecular methods [21] [22] and further confirm clonality of new O3:K6 and its serovariants.

### Pan-genome analysis

Comparative analysis of nine newly sequenced genomes and six publicly available genomes of *V. parahaemolyticus* could help us determine the global gene repertoire of the species. This can be described by its ‘pan-genome’ that includes a core genome containing genes present in all strains and a dispensable genome composed of genes absent from one or more strains and genes that are unique to some strains. Here, the core and pan-genomes of *V. parahaemolyticus* were identified using OrthoMCL [23].

**Figure 2:**
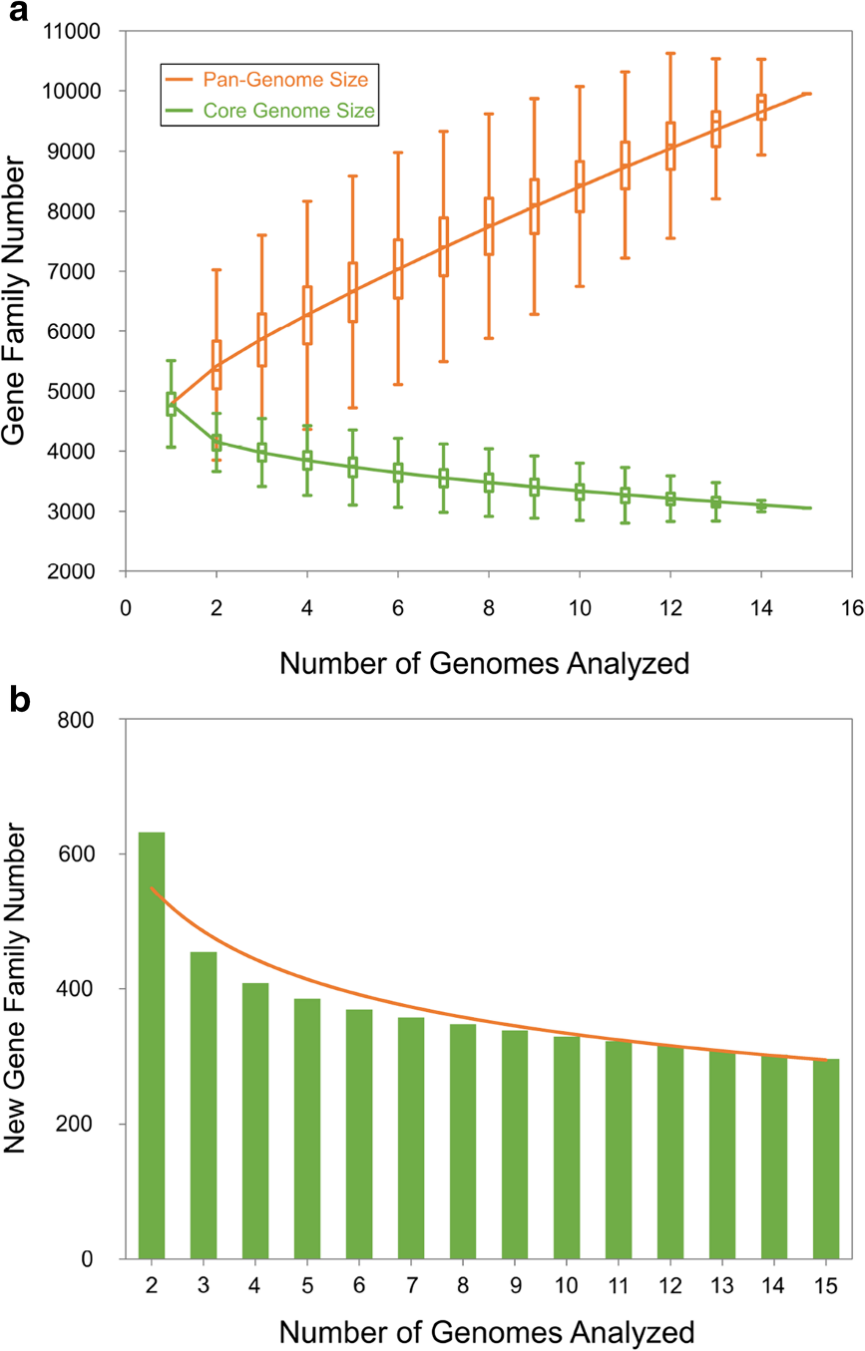
**Gene repertoire analysis of** ***V. parahaemolyticus.*** **(A)** Pan-genome and core genome size accumulation. **(B)** New gene family accumulation. The green bars indicate the number of expected new gene family detected for a particular number of genomes analysed, and the orange line indicates the trend of expected new gene family with an increasing number of genomes.

Our result shows that the number of pan-genome gene families increased with the number of genomes analysed, indicating that *V. parahaemolyticus* harbors an open pan-genome (Figure 2). We have also analyzed the trend of new gene families, and we found that new genes will continue to be found with increasing of much more genomes.

### Gene-content analysis of *V. parahaemolyticus*

**Figure 3:**
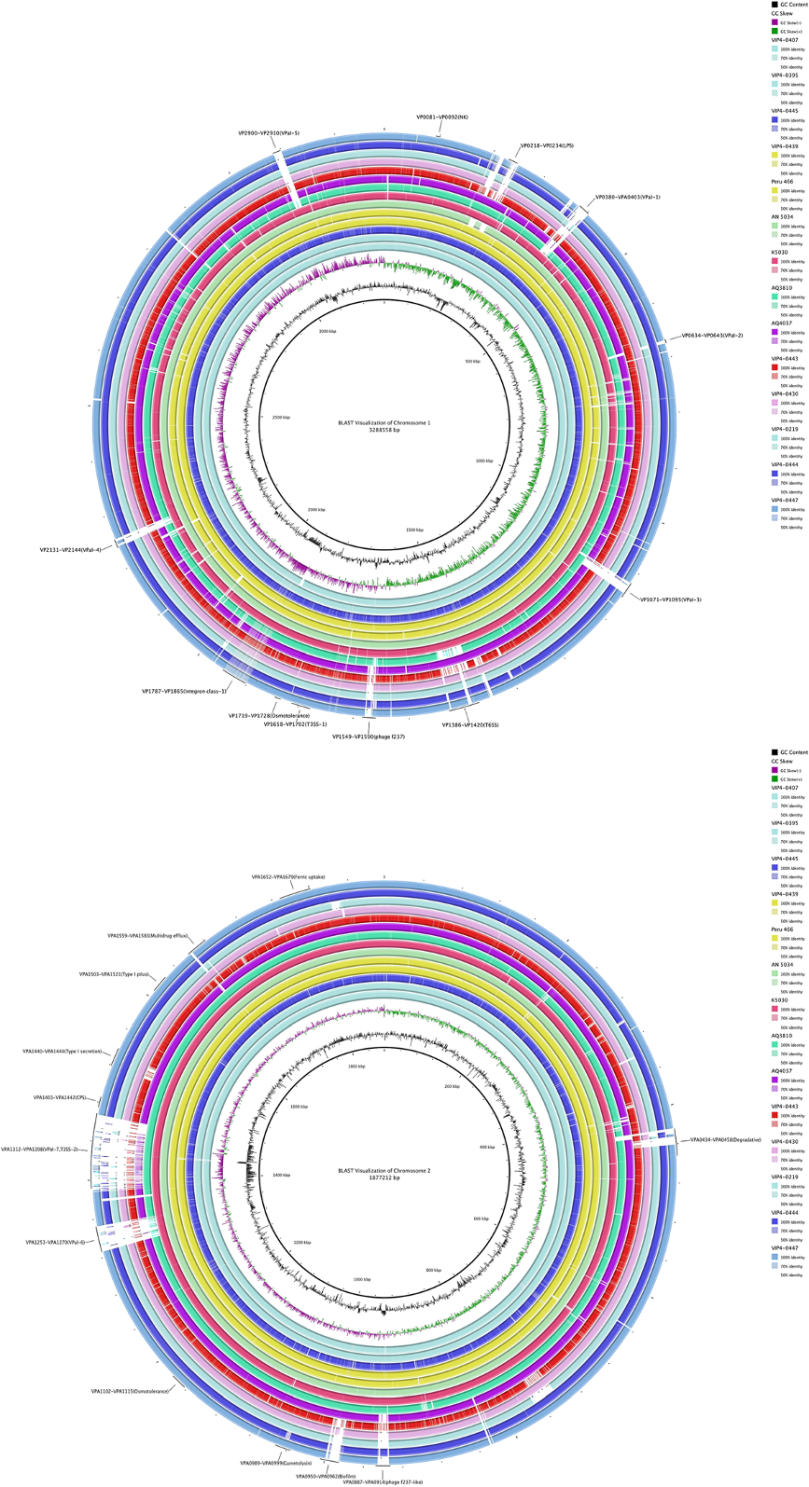
**BRIG visualization of** ***V. parahaemolyticus*** **genomes.** The innermost circles represent the reference sequence of *V. parahaemolyticus* 2210633. Outer rings illustrate shared identity with other *V. parahaemolyticus* isolates.

By investigating the presence and absence of genes in *V. parahaemolyticus*, we found that most regions within their genomes were conserved (Figure 3). We then further analyzed 24 genomic regions that are unique to *V. parahaemolyticus* RIMD 2210633 [24], which mainly include two T3SS, seven pathogenic islands, and f237 prophages. Comparative genomics revealed that these 24 regions accounted for most of the varying regions. Five pathogenic islands (VPal-1, VPal-3, VPal-4, VPal-5, and VPal-6) were found absent in not only the environmental strains, but also two pre-pandemic clinical strains. VPal-7 was found absent in AQ4037 and other environmental strains.

Previously, microarray-based approach failed to detect genes responsible for pathogenesis of *V. parahaemolyticus* [25]. Traditional features also failed to differentiate clinical strains from environmental strains [26, 27]. Here, analysis of these varying regions revealed the presence of only 11 regions in the clinical group, which were absent in the environmental group (Table 2). As expected, genes in T3SS2 were not found in all of the clinical strains, which further confirm that it is not reliable to simply use *tdh*, *trh* or genes in T3SS as virulence features [25]. However, a region located in chromosome II (984909–999901) contained genes from VPA0950 to VPA0957, which encode putative biofilm associated proteins and outer membrane proteins. A few genes including two type IV pili were previously described in biofilm formation of *V. parahaemolyticus*, however details of the process are still unexplored in this bacterium [28, 29]. In other *Vibrio* species such as *V. fischeri*, biofilm formation was found to play an important role in host colonization [30]. It is suggested that gene clusters found in clinical strains of *V. parahaemolyticus* could also provide this bacterium with advantages in host adaptation. These gene clusters have not been found previously, and we suggest that they could act as new candidate virulence markers.

**Table 2:** **Genomic regions that found in clinical group only**

Reference No.	Chr	Start	End	Length	Features in this regions*	Descriptions
NC_004603.1	Chr1	1630527	1631678	1151	VP1517, VP1518	Rhs-family protein
NC_004603.1	Chr1	1632125	1632898	773	VP1520, VP1521	Hypothetical protein
NC_004605.1	Chr2	433304	434859	1555	VPA0442,VPA0443	Hypothetical protein
NC_004605.1	Chr2	436019	437350	1331	VPA0445, VPA0446	Methylamine utilization protein MauG precursor
NC_004605.1	Chr2	438755	439983	1228	VPA0447	Hypothetical protein
NC_004605.1	Chr2	823596	824400	804	VPA0796	L-allo-threonine aldolase
NC_004605.1	Chr2	984909	986361	1452	VPA0950, VPA0951	Hypothetical protein
NC_004605.1	Chr2	987912	990219	2307	VPA0952, VPA0953(partial)	Biofilm-associated surface protein
NC_004605.1	Chr2	994472	995346	874	VPA0953 (partial)	Biofilm-associated surface protein
NC_004605.1	Chr2	995407	998918	3511	VPA0954, VPA0955, VPA0956, VPA0957 (partial)	Agglutination protein,outer membrane protein
NC_004605.1	Chr2	998994	999901	907	VPA0957 (partial)	Transporter binding protein

*The gene name is based on annotation of *Vibrio parahaemolyticus* RIMD 2210633.

### A novel genomic island commonly found in clinical strains

O3:K59 is a new serovariant of serotype O3:K6. It is recently reported that it could replace local serovariants and has caused pandemics in Chile and Peru [31, 32]. Here we analyzed the genome of our O3:K59 strain VIP4-0407 and found a novel genomic island that is absent in *V. parahaemolyticus* RIMD 2210633 [33]. This genomic island was found in most of our clinical strains including AQ4037, K5030, AN-5034, Peru-466, VIP4-0439, VIP4-0395, VIP4-0407 and AQ3810, but was absent in our environmental strains. We defined this genomic island as *Vibrio parahaemolyticus* island-8 (VPal-8). It contained six CDSs (Table 3), one of which showing high similarity with an AraC family transcriptional regulator that caused virulence in a mouse infection model in *Mycobacterium tuberculosis* [34]. ArcC family transcriptional regulators could regulate T3SS genes to modulate bacterial virulence [35], We analyzed sequences in this genomic island to determine whether there is any T3SS secreted protein. Using a combination of available T3SS prediction tools including T3_MM [36], Effective T4 [37], and T3SS effector prediction [38], we found that the third protein in this genomic island showed positive results in all of the prediction tools.

**Table 3:** **Gene clusters in small genomic island found in** ***V. parahaemolyticus***

No.	Accession ID	Start	End	No. of aa*	Homolog accession no.	Description
1	AXNM01000034	96777	97262	162	NP_797023.1	SsrA-binding protein
2	AXNM01000034	97884	99086	401	ZP_05776346.1	Phage integrase family protein
3	AXNM01000034	99645	100427	261	ZP_05888734.2	Putative cyclic diguanylate phosphodiesterase (EAL) domain protein
4	AXNM01000034	100420	101163	248	WP_025628220.1	AraC family transcriptional regulator
5	AXNM01000034	102145	101300	282	ZP_01990379.1	Hypothetical protein
6	AXNM01000034	103426	104361	312	ZP_01990406.1	Hypothetical protein

*Amino acids.

We further investigated whether VPal-8 is widespread across the population of clinical strains. Using this genomic island as a probe to perform southern hybridization on an additional population of 40 clinical strains, we found that this novel genomic island can be found in 29 of the clinical strains (Additional file 1: Table S1). Therefore, we suggest that the genomic island found in this study may be related to the virulence of *V. parahaemolyticus*.

### sRNAs and CRISPR element analysis

CRISPRs (Clustered regularly interspaced short palindromic repeats) could play important roles in the interaction of bacteria and mobile genetic elements [39]. We annotated CRISPR elements in *V. parahaemolyticus* using CRISPR finder [40] and found a total of six CRISPR elements (Table 4). Our environmental strains were found to harbor fewer CRISPR types, whereas at least two CRISPRs were found in each of the clinical strains.

**Table 4:** **Distribution of CRISPRs in** ***V. parahaemolyticus*** **strains**

Strain	Source	CRISPR 1	CRISPR 2	CRISPR 3	CRISPR 4	CRISPR 5	CRISPR 6
RIMD 2210633	Clinical	+	-	+	-	-	-
AQ3810	Clinical	-	+	+	-	-	+
Peru 466	Clinical	+	+	-	-	-	-
K5030	Clinical	+	-	+	-	-	-
AQ4037	Clinical	-	-	+	-	+	-
AN-5034	Clinical	+	-	+	-	-	-
VIP4-0439	Clinical	+	-	+	+	-	-
VIP4-0395	Clinical	+	-	+	-	-	-
VIP4-0407	Clinical	+	+	-	-	-	-
VIP4-0445	Clinical	+	+	-	-	-	-
VIP4-0444	Environmental	-	+	-	-	+	-
VIP4-0219	Environmental	-	-	+	-	-	-
VIP4-0447	Environmental	-	-	-	-	-	-
VIP4-0430	Environmental	-	-	-	-	-	-
VIP4-0443	Environmental	-	-	-	-	-	-

sRNAs are a class of non-coding RNAs in bacteria and are important post-transcriptional regulators in multiple crucial biological processes such as biofilm formation, quorum sensing and virulence [41, 42] . We determined the sRNA sequences in our *V. parahaemolyticus* strains and found that most sRNAs were highly conserved (Additional file 2: Table S2). However, a few sRNAs such as GcvB and STnc1460 were only present in a few strains, indicating that these sRNAs may mediate some strain-specific regulations.

### Biphenyl degradation pathway identified in environmental strains

**Figure 4:**
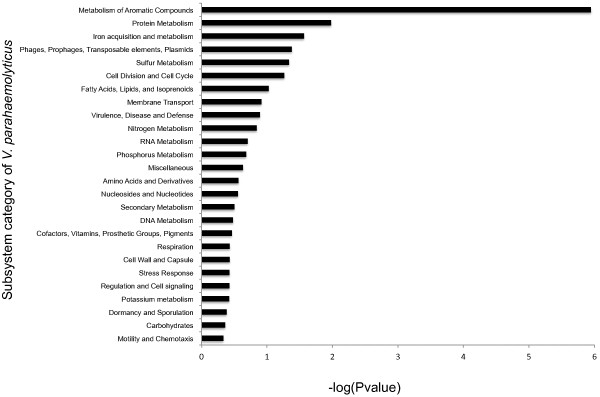
**Subsystem annotation of** ***V. parahaemolyticus.*** The y-axis represents subsystems annotated in the RAST server whereas the x-axis shows the respective -log(P) values. P-values were calculated to examine if there is significant difference in the numbers between the subsystems of environmental strains and clinical strains. The subsystem “metabolism of aromatic compounds” was shown to have a most significant P-value (P-value <0.05, −logP >1.3).

We annotated the subsystems of *V. parahaemolyticus* genomes using the RAST Server [43] (Figure 4). Five subsystems were found enriched in the environmental strains, including Metabolism of Aromatic Compounds (p = 1.12E06), Protein metabolism (p = 0.01), Iron acquisition and metabolism (p = 0.027), Phages and Prophages (p = 0.041) and Sulfur Metabolism (p = 0.046). The most enriched subsystem “Metabolism of Aromatic Compounds” contained an average of six genes in clinical strains, but an average of 10 genes in the environmental strains.

We further examined this category to investigate which pathway has contributed most of the differences. Genes involved in biphenyl degradation pathway (BphE1, BphC, Bphj2) were found in all of our environmental strains but not in the clinical strains, except the pre-pandemic strain AQ3810 (Table 5). The presence of the unique biphenyl degradation pathway in our environmental strains is possibly caused by the widespread of polychlorinated biphenyls in the environment [44].

**Table 5:** **Detection of biphenyl degradation pathway genes**

Strains*	Source	BphE1	BphC	Bphj2
VIP4-0444	Environmental	+	+	+
VIP4-0219	Environmental	+	+	+
VIP4-0430	Environmental	+	+	+
VIP4-0443	Environmental	+	+	+
VIP4-0447	Environmental	+	+	+
AQ3810	Clinical	+	+	+

*Only strains with positive results are shown here.

### Virulence potential of environmental strains

*V. parahaemolyticus* harbors many virulence factors including TDH, TRH and two T3SSs, which are generally pathogenic. Here we report the distribution of virulence factors and prophages in the genomes of our *V. parahaemolyticus* strains to reveal their pathogenic potential.

We predicted prophage regions in *V. parahaemolyticus* using the PHAST webserver [45] and found that clinical strains were restricted to a few prophage elements. The pandemic group of clinical strains possessed a filamentous prophage f237, which could increase virulence by adhering to intestinal cells [46]. A similar prophage was also found in the AQ3047 strain. In contrast, many diverse prophage elements were found in our environmental strains (Table 6). Six new prophages were found in four of our environmental strains, which harbor genes that could enhance the fitness for survival in specific environments. We further examined the relationship between the number of prophages and CRISPR in our strains. We analyzed the spacer sequences of CRISPRs and eight unique spacers sequence were found. However, we could not find any homologous prophage regions that could match with these spacer sequences in the respective strains. This could be explained by the possibility that the CRISPRs of *Vibrio parahaemolyticus* could inhibit the prophage insertion to the host genome. Indeed, an inverse correlation of CRISPRs with the number of prophage was also described in *Streptococcus pyogenes* [47].

**Table 6:** **Distribution of prophages in** ***V. parahaemolyticus*** **strains**

Strains*	Source	Prophage 1 (f237)	Prophage 2 (f237-like)	Prophage 3	Prophage 4	Prophage 5	Prophage 6	Prophage 7	Prophage 8
RIMD 2210633	Clinical	+							
Peru 466	Clinical	+							
K5030	Clinical	+							
AN-5034	Clinical	+							
VIP4-0439	Clinical	+							
VIP4-0395	Clinical	+							
VIP4-0407	Clinical	+							
VIP4-0445	Clinical	+							
AQ4037	Clinical	-	+						
VIP4-0444	Environmental	-		19312/19312	8265/8265				
VIP4-0447	Environmental	-		12018/19312	6500/8265	91629/91629			
VIP4-0219	Environmental	-					34088/34088		
VIP4-0430	Environmental	-						58355/58355	48569/48569

*VIP4-0443,AQ3810 was excluded because no prophage was found in this strain. Numbers denote the length of homolog sequences / the length of intact prophage.

*toxR* gene, located next to *toxS* gene, involves in the regulation of many virulence-associated genes of *V. parahaemolyticus* [48] *.* Variation in the *toxR* sequence can be used to differentiate phylogenetically distinct clusters in *V. parahaemolyticus*. Seven base positions were previously identified to distinguish the O3:K6 isolates before 1995 from the new O3:K6 clones within a 1346-bp region [49]. We further investigated the variations between these base positions within our *V. parahaemolyticus* isolates. In all of the seven new O3:K6 clones, the bases were perfectly mapped to the reference ones (Table 7). However, another environmental strain (VIP4-0443) also had the same sequence bases with the new O3:K6 clones, suggesting that it may also harbor virulence potential. We then further examined whether there are any other virulence proteins in this strain. A total of 264 candidate virulence genes were found (Additional file 3: Table S3), which further confirm that this environmental strain could have virulence potential.

**Table 7:** **Base variations in the** ***toxRS*** **sequence of selected** ***V. parahaemolyticus*** **strains**

Strains	Sources	Position at the ***toxRS***sequences						
		576	900	1002	1196	1214	1244	1463
RIMD 2210633	Clinical	A	A	T	T	T	A	T
Peru 466	Clinical	A	A	T	T	T	A	T
K5030	Clinical	A	A	T	T	T	A	T
AN-5034	Clinical	A	A	T	T	T	A	T
VIP4-0439	Clinical	A	A	T	T	T	A	T
VIP4-0395	Clinical	A	A	T	T	T	A	T
VIP4-0407	Clinical	A	A	T	T	T	A	T
VIP4-0445	Clinical	A	A	T	T	T	A	T
AQ4037	Clinical	G	G	C	C	A	G	A
AQ3810	Clinical	G	G	C	C	A	G	A
VIP4-0444	Environmental	G	G	C	C	A	G	A
VIP4-0447	Environmental	G	G	C	C	A	G	A
VIP4-0219	Environmental	G	G	C	C	A	G	A
VIP4-0430	Environmental	G	G	C	C	A	G	A
VIP4-0443	Environmental	A	A	T	T	T	A	T

### Construction of a genome sequence database for *V. parahaemolyticus*

**Figure 5:**
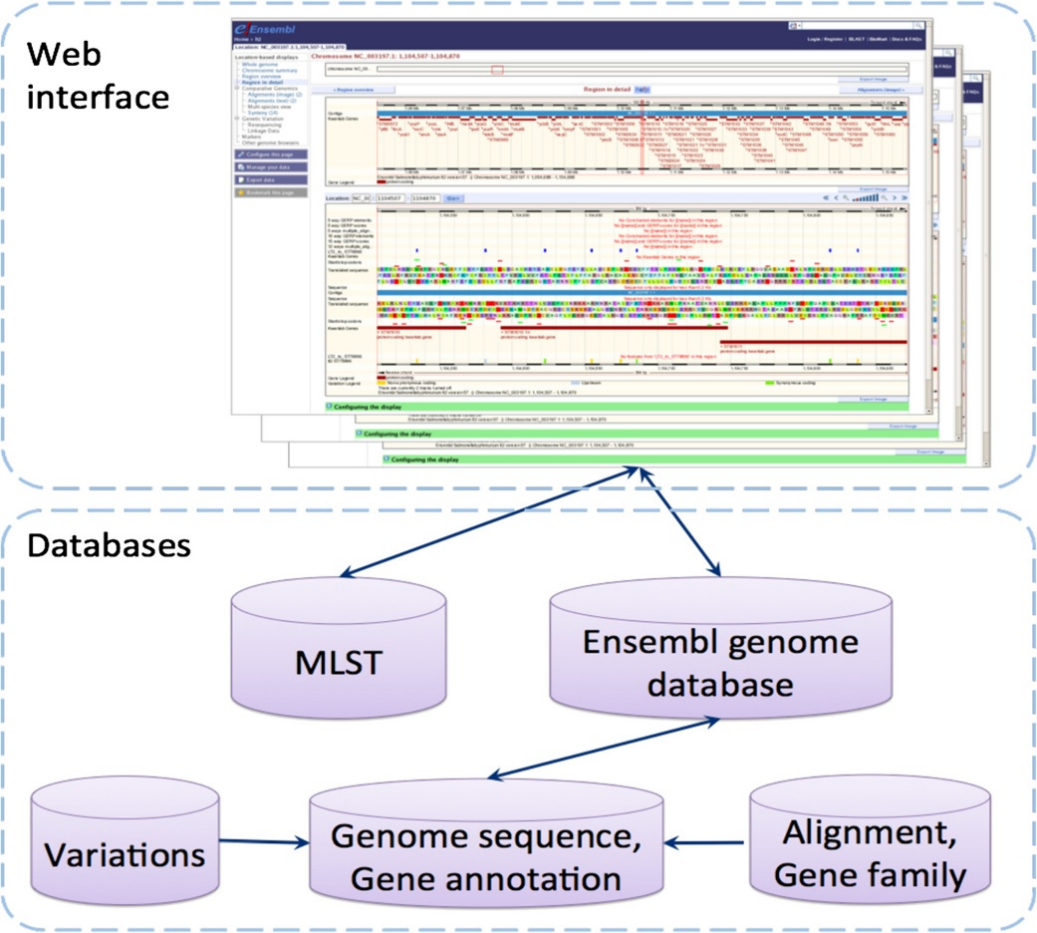
**Architecture of the Ensembl-based genome database for** ***V. parahaemolyticus.***

A *V. parahaemolyticus* genome database was constructed here based on the Ensembl genome annotation system using Perl scripts (Figure 5). Genome DNA sequences can be input into the platform via a user-friendly web-based interface. Our web-based database allows comparative analysis and data mining using available *V. parahaemolyticus* genome data. A suite of useful computational tools is now available for data analysis such as MLST and detection of genetic variations. The web-based database is now launched as a publicly accessible domain (http://kwanlab.bio.cuhk.edu.hk/vp).

## Conclusions

This is the first study on the genome dynamics of *V. parahaemolyticus* at the species level. We have three main conclusions. First, by comparing the genome sequences, we found that our clinical strains were phylogenetically distinct from the environmental strains. We discovered a new gene cluster belonging to the biofilm associated proteins of *V. parahaemolyticus*. We suggest that this novel gene cluster is a new virulence marker differentiating clinical from environmental strains. We also found a novel genomic island (VPal-8) that frequently distributed in the clincial strains. Second, by analyzing the virulence features and prophage elements of our environmental strains on a genome-wide scale, we discovered a new type of *toxRS* in one of the environmental strains and a diverse array of prophage elements in the environmental strains. These results indicate that the environmental strains studied could have virulence potential. Third, we set up an Ensembl-based genome database for *V. parahaemolyticus* to provide a unified and user-friendly platform to access all currently available genome sequences and annotations of this bacterium.

## Methods

### Ethics statement and isolate collection

Nine isolates of *V. parahaemolyticus* were selected from the bacterial archive maintained at the microbiology laboratory of the Department of Health of Hong Kong and were characterized for genome sequencing. The study was approved by the Joint CUHK-NTEC Clinical Research Ethical Committee at the Prince of Wales Hospital in Hong Kong. Written informed consent was obtained from all the studied subjects for sample collection and subsequent analysis.

### DNA sequencing

Whole-genome shotgun sequencing was performed using a 454 Genome Sequencer (GS) FLX-Titanium (Roche Diagnostics, US). DNA of each selected isolate was extracted from culture and subjected to sequencing library preparation according to the manufacturer’s recommended protocols. Libraries of processed genomic DNA fragments immobilized on DNA capture beads were individually sequenced on a PicoTiterPlate device. Bases sequenced and the corresponding quality values were called and delivered in a standard format by GS-FLX-Titanium system for downstream bioinformatic analyses. The remaining genome gaps were filled by the GS-FLX-provided paired-end approach and/or primer walking. The primer walking approach involved design of primers flanking the gaps and PCR to amplify the DNA fragments covering the gaps. PCR products were then directly sequenced using the ABI DNA Sequencer (Applied Biosystems, US).

### Sequence assembly and annotation

Raw sequence reads were first filtered to remove low-quality reads. Sequence assembly was performed on the remaining clean reads *de novo* using the GS Newbler 2.7. The genome sequences were annotated using the RAST webserver. Contigs were reordered and genome comparisons were carried out using mauve program [50]. BRIG was used for genome alignment visualization [51]. Prophage elements of *V. parahaemolyticus* were predicted using PHAST [45].

### Phylogenetic tree construction

SNPs among our isolates and several foreign strains were identified using GS Reference Mapper (Roche Diagnostics). SNPs detected among all strains were concatenated together and a neighbor-joining phylogenetic tree was constructed using MEGA 6 [52] with 500 bootstrap pseudoreplicates. *Vibrio harveyi* ATCC BAA-1116 was used as outgroup for defining the root.

### Small RNAs and CRISPRs

A list of small RNA sequences was retrieved from the BSRD database [42]. sRNA homologs in our *V. parahaemolyticus* isolates were then identified using BLASTn. The e-value of BLAST was set to 1e-5. Annotation of CRISPR elements was done using CRISPRfinder [40].

### Experimental validation of genomic island

Two sets of primers were designed for PCR-based detection of genomic island in a population of clinical *V. parahaemolyticus*. These clinical strains were previously used for microarray-based analysis [53].

### Availability of supporting data

Data of this Whole Genome Shotgun project have been deposited at GenBank under the accessions AXNJ00000000, AXNK00000000, AXNL00000000, AXNM00000000, AXNO00000000, AXNP00000000, AXNQ00000000, AXNR00000000, and AXNS00000000. The phylogenetic tree and associated data matrix are available in TreeBASE database (Accession URL: http://purl.org/phylo/treebase/phylows/study/TB2:S16780).

## Electronic supplementary material

Additional file 1: Table S1: Primers used in PCR detection of genomic island in *V. parahaemolyticus* isolates. (XLSX 40 KB)

Additional file 2: Table S2: Distribution of sRNA sequences in *V. parahaemolyticus* strains. (XLSX 44 KB)

Additional file 3: Table S3: Homologous virulence-associated genes in environmental strain VIP4-0443. (XLSX 70 KB)
